# Shedding a New Light on Skin Aging, Iron- and Redox-Homeostasis and Emerging Natural Antioxidants

**DOI:** 10.3390/antiox11030471

**Published:** 2022-02-27

**Authors:** Charareh Pourzand, Andrea Albieri-Borges, Nico N. Raczek

**Affiliations:** 1 Medicines Design, Department of Pharmacy and Pharmacology, University of Bath, Bath BA2 7AY, UK; 2 Medicines Development, Centre for Therapeutic Innovation, University of Bath, Bath BA2 7AY, UK; 3 Research and Development, ASEA LLC., Pleasant Grove, UT 84062, USA; aborges@aseaglobal.com (A.A.-B.); nraczek@aseaglobal.com (N.N.R.)

**Keywords:** skin, redox, antioxidant, aging, photoaging, sunlight, photoprotection, anti-aging

## Abstract

Reactive oxygen species (ROS) are necessary for normal cell signaling and the antimicrobial defense of the skin. However excess production of ROS can disrupt the cellular redox balance and overwhelm the cellular antioxidant (AO) capacity, leading to oxidative stress. In the skin, oxidative stress plays a key role in driving both extrinsic and intrinsic aging. Sunlight exposure has also been a major contributor to extrinsic photoaging of the skin as its oxidising components disrupt both redox- and iron-homeostasis, promoting oxidative damage to skin cells and tissue constituents. Upon oxidative insults, the interplay between excess accumulation of ROS and redox-active labile iron (LI) and its detrimental consequences to the skin are often overlooked. In this review we have revisited the oxidative mechanisms underlying skin damage and aging by focussing on the concerted action of ROS and redox-active LI in the initiation and progression of intrinsic and extrinsic skin aging processes. Based on these, we propose to redefine the selection criteria for skin antiaging and photoprotective ingredients to include natural antioxidants (AOs) exhibiting robust redox–balancing and/or iron-chelating properties. This would promote the concept of natural-based or bio-inspired bifunctional anti-aging and photoprotective ingredients for skincare and sunscreen formulations with both AO and iron-chelating properties.

## 1. Introduction

Skin, as the largest organ in the body, is crucial for sensing and vitamin D synthesis. It also provides a major barrier for protecting the body against environmental chemical and physical assaults and pathogens, while insulating and regulating its temperature to avoid uncontrolled water and solutes loss [1,2]. This makes the skin the most exposed organ to environmental factors (i.e., exposomes) such as pollution, temperature, and sunlight radiations which, depending on the biological responses governed by various internal genetical and non-genetical factors, can contribute to both intrinsic and extrinsic aging of the skin [3].

Despite the knowledge of skin aging exposomes and the numerous studies on biological responses of human skin to such threats, the dual influences of skin redox- and iron-homeostasis in skin aging are mostly overlooked [4,5,6]. The intimate relationship between redox- and iron-homeostasis in the body implies that any external or internal factor that would disrupt redox homeostasis would inevitably create an imbalance in iron homeostasis.

**Figure 1:**
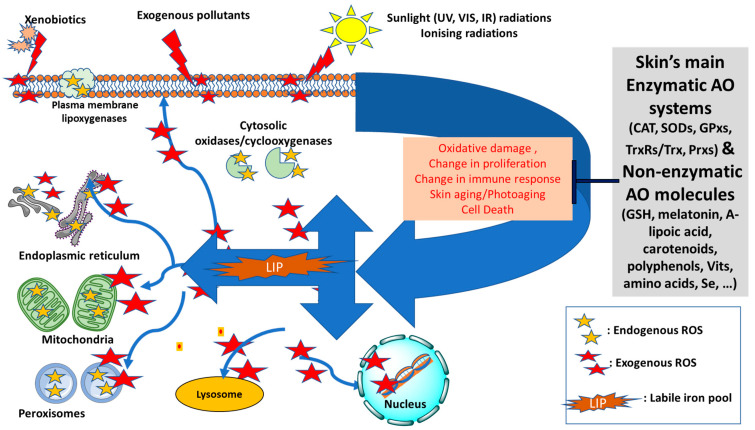
Diagram of the redox balance in the skin—Endogenous sources of reactive oxygen species (ROS, shown as yellow stars) can be generated intracellularly in mitochondria, peroxisomes, plasma membrane (lipoxygenases) or cytosolic enzymatic systems (oxidases and cyclooxygenases). The skin’s antioxidant (AO) defense will maintain the redox homeostasis by neutralising the excess ROS that are likely to form by exogenous factors, notably sunlight radiations, xenobiotics or pollution. The increase in the level of intracellular labile iron pool (LIP) disrupts the iron homeostasis and intensifies the oxidative damage to cell components due to formation of highly reactive oxygenated species via Fenton Chemistry (shown as red stars). As a result, the AO defense system can be overwhelmed and redox homeostasis disrupted leading to significant oxidative cell damage, change in cell proliferation and immune response, acceleration of aging processes and even cell death (Adapted and modified from references [4,5,6]). UV: ultraviolet; VIS: visible; IR: infrared; CAT: catalase; SODs: superoxide dismutases; GPxs: glutathione peroxidases; TrxRs/Trx: Thioredoxin reductases/Thioredoxin; Prxs: Peroxiredoxins; GSH: glutathione; Vits: vitamins.

At the cellular level, redox homeostasis is linked to reactive oxygen species (ROS) that are continuously produced in various cellular compartments as by-products of aerobic reactions for various cellular processes notably signal transduction, metabolism, proliferation, gene expression and the programmed cell death, apoptosis [7,8,9,10,11] (see Figure 1). Under normal conditions, low levels of ROS actively participate in redox reactions and may also act as second messengers for regulatory functions, as part of the redox homeostasis of the organism. However, under conditions of oxidative stress, excess production of ROS can be detrimental for the cells and body especially in the presence of high levels of harmful labil iron (LI). This is because LI can catalyse the production of highly reactive species such as hydroxyl radical (^•^OH) via the Fenton reaction or superoxide-driven Fenton chemistry [4,5,6]. The LI-catalysed ROS can severely damage cell constitutents and exacerbate the oxidative damage that is already occurring in the cells, as observed for example after solar ultraviolet A (UVA, 320–400 nm) irradiation in skin cells [12,13].

Due to the dangerous nature of LI, the intracellular iron homeostasis is tighly controlled by iron regulatory proteins (IRPs) that post-transcripionally regulate the levels of proteins involved in LI’s uptake (transferrin receptors 1 and 2), storage (ferritin) and export (ferroportin) to minimise its harmful levels in the cells [5,14]. However, under conditions of oxidative stress, the inevitable disruption of iron (and redox) homeostasis can lead to oxidative damage, aging and numerous pathologies [15,16].

There are two aspects that link iron homeostasis to skin aging. Firstly, during chronological aging, iron accumulates in the body, notably in the skin [5,17]. In women, this accumulation is more pronounced after menopause when the iron excreting route of menstruation stops. The presence of high iron in post-menopausal women has been linked to increased LI-catalysed oxidative damage in the skin, contributing to the acceleration of the aging process in the skin [5,17,18,19]. Secondly, the constant exposure of skin to oxygen and the oxidising component of sunlight, notably UVA radiation (320–400 nm), is detrimental, especially due to the high content of iron and ferritin of the aged skin [5,12,13,17,18,20]. This is because UVA can promote the immediate proteolytic degradation of ferritin in skin cells, leading to an increase in the intracellular level of potentially harmful LI contributing to iron dyshomeostasis, which in turn exacerbates the redox imbalance due to an LI- catalysed burst of ROS [13,14,20]. In addition to ROS-mediated effects, oxidative phosphorylation in the mitochondria can also act as a major contributor to the overall process of intrinsic aging [21,22,23]. Moreover, the presence of a high concentration of redox active LI in the mitochondria capable of participating in the Fenton reaction make these organelles particularly sensitive to oxidative stress, notably the UVA component of sunlight leading to photosensitivity, photodamage, and photoaging [5,24,25].

Therefore, the understanding of the mechanisms underlying the redox- and iron-homeostasis disturbances by UVA and other environmental oxidising insults in relation to skin aging appears to be crucial for devising skin anti-aging and photoprotective strategies. Significantly, while the consequences of alterations of both redox- and iron-homeostasis of the skin that occur in intrinsic chronological aging and even more in extrinsic photoaging are well-documented [4,5,6,26,27], there is no effort to address the detrimental role of LI when developing skin anti-aging and photoprotective products.

**Figure 2:**
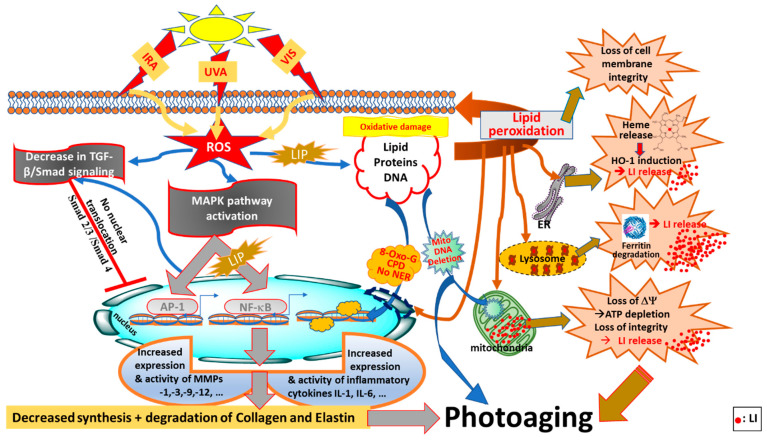
Photoaging pathways through disruption of redox- and iron-homeostasis by oxidising components of sunlight (UVA, VIS, IRA). ROS (reactive oxygen species) generated by oxidising components of sunlight, notably UVA, can activate the MAPK (mitogen-activated protein kinase) and NF-κB (nuclear factor kappa B) signaling pathways, as well as AP-1 (activator protein 1) and NF-κB transcription factors leading to increased expression of proinflammatory cytokines (e.g., Interleukins IL-1β and IL-6) and MMPs (matrix metalloproteinases) that contribute to photoaging by changing concomitantly the composition of ECM (extracellular matrix) components by breaking down the collagen and elastin networks and regulating the TGF-β/Smad (tumour growth factor-B/Smad) signaling pathway to reduce collagen production. UVA-induced increase in LIP (labile iron pool) is also a major contributor to NF-κB activation and the related inflammatory response. Concomitantly with this, ROS generated by oxidising components of sunlight can regulate the TGF-β/Smad (tumour growth factor-B/Smad) signaling pathway to reduce collagen production, and ultimately accelerate skin photoaging. The presence of LIP in different compartments of cells, notably cytosol, lysosomes and mitochondria, makes the skin cell constitutents (i.e., lipid, protein and DNA) highly vulnerable to iron-catalysed ROS damage leading to lipid peroxidation, oxidative DNA 8-oxo-G (8-hydroxy guoanosine adducts) and CPDs (cyclopyrimidine dimers), and for mitochondrial DNA as deletions and finally for proteins as oxidation and degradation. The UVA-induced increase in cytosolic LIP also occurs as a result of proteolytic degradation of ferritin and release of heme from ER (endoplasmic reticulum) and activation of HO-1 (heme-oxygenase 1), all of which disrupt the cellular redox (and iron) homeostasis, leading to photoaging (taken and modified from references [5,78,112,113,114]).

In this review, we have shed a new light on redox balance in the skin by highlighting how the pathways of both endogenous and exogenous ROS productions are linked to intracellular LI levels. In addition, we have highlighted the neglected pathways by which, under conditions of oxidative stress, the increase in endogenous LI can cause abrupt disruptions to both iron and redox homemostasis, intensifying the ongoing oxidative damage to skin cell components (see Figure 1) [10,11]. After summarising the key features differentiating between ‘intrinsic’ (chronological) skin aging and ‘extrinsic’ skin photoaging, we have introduced the interplay between redox- and iron-homeostasis in photoaging processes (see Figure 2). In the latter, we have introduced for the first time a comprehensive summary of the multiple pathways by which the concomitant increase in LI levels and ROS production contribute to the photodamage and photoaging processes. We have also introduced the major antioxidant (AO) systems of the skin and discussed their limited capacity under the conditions of oxidative stress in the presence of excess LI and accompanied ROS levels. Using this knowledge, we have then highlighted the need for robust natural-based products with either strong redox-balancing properties or bifunctional products with both AO and iron-chelating properties to overcome the oxidative stress conditions in the skin, with some promissing examples provided.

## 2. ‘Intrinsic’ (Chronological) Skin Aging versus ‘Extrinsic’ Skin Photoaging

Aging affects the functional role of the skin, notably its protection against physicochemical and biological attacks, as well as its thermoregulatory, sensory, immunological, and hormonal functions [28]. Human skin can experience both intrinsic (chronological) and extrinsic aging (i.e., photoaging) [29]. The latter occurs as a result of environmental damage, notably sun-induced photodamage leading to photoaging. Both photoaging and chronological aging processes are cumulative [30]. However, unlike chronological aging, which is mostly time-dependent, photoaging may be a premature process depending on the frequency and duration of sun exposure throughout the life of the individuals. In addition, the outdoor lifestyles of the individuals, especially when living in sunny climates, may accelerate the premature photoaging of their skin. Furthermore, the skin type and the level of melanisation would predict the predisposition of individuals to sunlight-mediated photodamage and photoaging [31,32].

Both photoaging and chronological aging processes affect the epidermal and dermal layers of the skin. Histologically, chronological aging shows excessive epidermal thinning accompanied by a loss of hydration and wrinkles. The epidermal thinning is due to a gradual decrease in the proliferation of the epidermal basal layer cells leading to a decreased contact surface area between the dermis and epidermis, resulting in a smaller exchange surface for dermal nutrition supply to the epidermis [33]. This phenomenon is part of cellular senescence in which the proliferative ability of skin cells (i.e., fibroblasts, keratinocytes, and melanocytes) gradually declines [23].

In contrast to epidermal thinning in chronological aging, photoaged skin is associated with a thick and leathery texture as well as deeper wrinkles and uneven pigmentation [34]. These changes are due to an accumulation of a significant amount of elastotic material in the photoaged dermis via a process called solar elastosis. Although solar elastosis is not a typical feature of chronological skin aging, the gradual modification of the elastin network has been observed in sun-protected skin. The elastin fibres are usually thin and single-stranded in the young skin. However, as the skin ages, they become progressively encrusted with the disappearance of terminal fibrils extension into the epidermis [35]. Moreover, the assembly of protein constituents of elastin fibres declines during chronological skin aging [36]. From a histological point of view, in both types of skin aging, the modifications observed in dermal connective tissue are more pronounced than the cornified envelope [37,38]. For example, the chronological aging of the skin provokes the fragmentation of collagen fibrils and a significant decline in the collagen production that together weakens the skin constituency and makes it prone to bruising [31,39,40,41,42]. From a molecular point of view, the transcription factor, nuclear factor erythroid 2-related factor 2 (Nrf2), is the master regulator of the AO response protecting skin cells against oxidative insults. Various natural and synthetic compounds, particularly electrophiles, having abilities to activate Nrf2-regulated AO defense and promote redox balance, have been demonstrated to provide promising photoprotective effects against skin damage [43].

The fragile tissue microenvironment occurring in chronological aging as a result of modifications of the extracellular matrix (ECM) and impairment of the dermal structure and mechanical properties is similar to features observed in age-related skin disorders. These include atrophy, reduced tensile strength, loss of hydration and impaired vasculature support [44,45]. In addition, the aged-related modifications of genetic material in the skin as well as its stromal microenvironment have been linked to the development of cancer [46]. The regulation of collagen degradation is usually carried out by matrix metalloproteinases (MMPs) and their natural inhibitors called tissue inhibitors of metalloproteinases (TIMPs). MMPs are zinc-containing endopeptidases that are capable of degrading a wide range of ECM proteins [42]. Under normal conditions, collagen turnover in human skin is carried out by MMP-1, -8, -13 and -14. However, in an aged skin counterpart, the initiation of collagen breakdown is mostly mediated by MMP-1 [47,48].

Mitochondrial dysfunction is also recognised as one of the hallmarks of skin aging, with mitochondrial DNA (mDNA) deletions, excess ROS accumulation and oxidative stress observed in both epidermal and dermal skin compartments [28,49,50]. On the other hand, it is suggested that age-associated MMP-1 expression is redox-sensitive and the redox-dependence is initially due to the activation of the c-Jun N-terminal kinase (JNK) pathway. In this way, JNK is one of the key mediators of redox dependent MMP-1 induction. The senescence-dependent MMP-1 induction is a complex signaling process dependent on ROS regulating a number of distinct signaling networks that converge to drive MMP-1 expression [51].

### 2.1. Terrestrial Sunlight Radiations

The sun emits a wide range of electromagnetic energies which are known as the solar spectrum. The solar spectrum ranges from infrared (IR, 770 nm–1 mm) and visible (VIS, 400–770 nm) to ultraviolet (UV, 100–400 nm) radiations. The IR range comprises 50% of the total energy of the sun emission that reaches the skin of individuals. The UV component of sunlight only represents about 5% of the incident radiation and is subdivided into UVA (320–400 nm), UVB (290–320 nm) and UVC (100–290 nm) wavelengths. UVC is stopped by the ozone layer, so the terrestrial UV radiation (UVR) is composed of UVA and UVB, with the majority being UVA (>95%). UVA consists of UVA1 (340–400 nm) and UVA2 (320–340 nm) in a 75% to 25% ratio. Until recently, the general assumption was that UVB and UVA are the main radiations responsible for the harmful effects of sunlight on human skin. As a result, the majority of photobiological studies on human skin have concentrated on the UV spectrum and especially on shortwave UVB wavelengths [52]. However, there is now a growing body of evidence suggesting that both VIS and near infrared (IRA, 770–1400 nm) spectral range of radiations are also capable of damaging the human skin. These findings have influenced the skin photoprotection strategies, with novel sunscreens and skin care products in the market claiming to possess protection against solar VIS and IRA spectral regions [52,53,54]. Nevertheless, the spectral region of UVA1/VIS (375–415 nm) is still being neglected and most of current sunscreens lack appropriate protection against this region of sunlight [55]. Furthermore, it is worthy of note that the sun protection factor (SPF) primarily measures protection against the UVB component of sunlight which has been shown to be much more erythemogenic than UVA radiation [56]. While the importance of UVA radiation in sun photoprotection is increasingly recognised, unlike SPF, the UVA protection factor (UVA-PF) definition still very much depends on the regulatory domains used [57].

### 2.2. Photodamaging Effects of Solar UVA and VIS Radiations

The major targets for terrestrial solar UV in humans are the skin and the eyes. The transmission of UV through these tissues and cells increases with the wavelength as a result of reduced molecular absorption [58,59,60,61]. In the skin, UVB, and to a greater extent UVA radiation, reach targets deep below the surface of the skin. Up to 35–50% of the overall UVA component of sunlight can reach the dermis in Caucasian skin [62], while a small but physiologically relevant proportion of long wavelength UVA can be absorbed by blood components [60]. The biological effects of UVA and UVB are dictated by the type of biomolecules they interact with. UVA is oxidative in nature and generates ROS notably ^1^O_2_, H_2_O_2_, O_2_^•−^ and ^•^OH in exposed cells and tissues indirectly through its interaction with intracellular chromophores. The most important ROS generated intracellularly by UVA are ^1^O_2_ and H_2_O_2_ with the ability to induce biological damage in exposed tissues via iron-catalysed oxidative reactions, since UVA also promotes an immediate increase in the cytosolic pool of LI [12,63,64,65,66,67,68,69]. Due to its dual effect as ROS generator and LI enhancer, UVA is referred to as the main oxidising component of sunlight [70,71]. Recent studies have highlighted that UVA can compromise DNA repair in human cells, and it does this by damaging DNA repair proteins, some of which are redox-regulated [72,73]. In addition, UVA radiation is capable of inducing both direct and indirect damage to DNA, as well as inhibiting DNA repair mechanisms leading to the initiation of skin carcinogenesis. While UVB does have an oxidative component, it induces lesions into DNA and damages proteins mostly by direct absorption [60]. Therefore, both UVA and UVB components of sunlight are equally carcinogenic for the skin, especially in a fair-skinned population, and this notion should be taken into account while devising strategies for skin photoprotection [32,74,75,76,77].

Studies from Pourzand’s laboratory and others have demonstrated that the presence of high levels of redox active LI in lysosomes, mitochondria, microsomes and the nucleus of skin cells sensitises these organelles to UVA-induced oxidative damage, even at natural sun exposure levels [5,13,78,79]. Damage to lysosomes causes the UVA-induced degradation of the cytosolic iron storage protein, ferritin by lysosomal proteases that are released to the cytosol [13]. The re-synthesis of ferritin following UVA irradiation takes several hours, so the harmful excess of cytosolic LI cannot be safely sequestered. Therefore, LI in conjunction with ROS generated during and after UVA irradiation, continues to exacerbate the iron-catalysed damage of irradiated skin cells, notably peroxidative damage in plasma membranes, resulting in a loss of cell membrane integrity and ultimately causing necrotic cell death [12,13,16,80]. UVA-mediated iron-catalysed damage to the mitochondrial membrane leads to a depletion of mitochondrial ATP, which is recognised as a hallmark of necrotic cell death [14].

In contrast to UVB, the biological effects of UVA radiation are strongly oxygen-dependent and can be modified by agents, which can be broadly classified as antioxidants (AOs). The skin contains a variety of small AO molecules, notably uric acid, ascorbate, sulfhydryls, ubiquinols, tocopherols, flavonoids, and carotenoids. In addition, a series of AO enzymes, which are also part of the redox system, notably glutathione peroxidases (GPxs), superoxide dismutases (SODs) and catalase (CAT) are also present in the skin cells and tissue [6] (see Section 3). UVA has the ability to decrease the cellular AO content of skin cells and tissue and this is thought to occur via the UVA-induced generation of ROS which depletes and damages both the cellular non-enzymatic and enzymatic AO defenses [60] (see Section 3).

At a low level of UVA insult, the skin’s AO molecules and enzymes provide some cellular defense against UVA-induced oxidative stress. However, upon exposure to high doses of UVA (e.g., during recreational summer holidays or occupational sun exposure of farmers and outdoor workers), the excess production of highly reactive ROS, notably ^•^OH (via a LI-catalysed Fenton reaction) are likely to overwhelm the cellular AO defence capacity of the skin, as well as disturbing the redox homeostasis, so the damaged cells may die by apoptosis or necrosis, depending on the severity of the insult. Alternatively, the damage may accumulate and be processed in such a manner that carcinogenesis is initiated or promoted [12,16]. The deleterious consequences of ROS-mediated and LI-catalysed oxidative damage by UVA have been shown to play a key role in skin photoaging and photocarcinogenesis [5,81]. Therefore, solar UVA can be considered as a major disruptor of both redox- and iron-homeostasis in the skin cells.

Studies from Pourzand and Tyrrell laboratories have shown that the UVA-induced increase in cytosolic LI is a key regulator of the nuclear factor kappa-light-chain-enhancer of activated B cells (NF-κB) [78]. The latter highlights the role of LI in skin inflammation caused by UVA. In this context, UVA is a strong activator of several genes involved in the inflammatory response, such as interleukins (ILs), intercellular adhesion molecule 1 (ICAM-1) and heme oxygenase 1 (HO-1). The therapeutic potential of HO-1 has been suggested in some studies, due to its cytoprotective and anti-inflammatory function against oxidative stress seen in pathologies or induced by environmental agents [82,83,84,85,86]. In addition, UVA is able to increase the level of both anti- and pro-oxidant enzymes such as manganese-dependent superoxide dismutase (Mn-SOD), glutathione peroxidase (GPx), NADPH-oxidase, ferritin and methionine-S-sulphoxide reductase with the consequent exacerbation or restoration of redox homeostasis [87].

The use of electron spin resonance (EPR) spectroscopy in skin biopsies demonstrated that half of the total skin’s oxidative burden is generated by VIS light. This is consistent with the ROS-generating capability of VIS light [88]. It has also been shown that VIS light is capable of inducing pigmentation in skin types IV and higher but not in type II [89]. The latter study suggests that VIS light may be a contributor to skin disorders such as melasma [71]. Although action spectra studies of DNA damage have established that while UVA1 is more DNA damaging than VIS light [90,91,92], the skin pigment melanin has been specifically shown to promote DNA damage in melanocytes exposed to VIS light, in the form of DNA strand breaks and oxidative pyrimidine modifications (but not cyclobutane pyrimidine dimers, CPDs). These effects have not been observed in the melanin- deficient (albino) equivalents [93].

The formation of ‘dark’ CPDs in melanocytes and pigmented skin appear to occur long after UVA irradiation via the chemo-excitation of melanin [94]. The same phenomenon has also been observed in melanin-deficient cells, suggesting that chromophores other than melanin can also undergo chemo-excitation [95]. Additional in vitro and in vivo studies have revealed that the UVA1/VIS boundary wavelengths promote significant DNA damage in the form of dark CPDs due to ROS generation via skin chromophores absorbing in these ranges. These results have important consequences for skin photoprotection and sunscreen testing protocols which, based on the above data, need to extend to wavelengths longer than 380 nm [52]. The spectral UVA1 and VIS regions of the sunlight have also been shown to generate NO^•^ which led to a reduction in blood pressure [96,97,98] and the regulation of pigmentation through Opsin-3 [99,100]. Opsin-3 is a member of the light-sensitive cell surface receptor family of opsins that plays an important role in photoageing of the skin. It is highly expressed in skin fibroblasts and regulates the UVA-mediated activation of MMPs (i.e., MMP1, MMP2, MMP3 and MMP9) via the calcium-dependent G protein-coupled signaling pathway [101].

### 2.3. Photodamaging Effects of Terrestrial IRA Radiation

The largest part of solar IR radiation is IRA (30% of total solar energy), which penetrates deeply into human skin, while IRB (mid IR, 1400–3000 nm) and IRC (far IR, 3000 nm–1 mm) mostly affect the upper skin layer [3,102]. Nearly 50% of the overall IRA component of sunlight can reach the dermal compartment of the human skin which is significantly higher than that of solar UVA radiation (i.e., 19%). Accordingly, the percentage of solar IRA radiation reaching the subcutaneous (i.e., 17%) tissue is significantly higher than that of the UVA component of sunlight (i.e., 1%). Similarly, to solar UVA radiation, the IRA component of sunlight reaches the subcutaneous compartment without markedly increasing the surface temperature of the skin. Compared to solar IRA, IRB radiation affects much less the dermal (20%) and subcutaneous layers of the skin tissue (8%), since it is mostly absorbed by the epidermal layer of the human skin (72%). Solar IRC on the other hand, affects neither the dermis nor the subcutaneous tissue as it is fully absorbed by the epidermal layer of the skin. This leads to an increase in temperature resulting in a skin sensation that can vary from pleasant warmth to thermal burn [58].

Solar IRA radiation has been shown to generate ROS in cultured skin cells and human skin (ex vivo and in vivo) [88,103,104]. IRA is strongly absorbed by mitochondrial components and the copper atoms of the complex IV of the mitochondrial respiratory chain which are considered as the major IRA chromophores. This is in agreement with the observation that fibroblasts irradiated with IRA exhibit increased ROS levels within their mitochondrial organelles [105]. As ROS have been shown to act as second messengers, this may be important for the gene expression mediated by IRA [63]. The fact that IRA pre-exposure can prevent the formation of UVB-induced sunburn cells provides a strong argument for its role in photocarcinogenesis [106,107].

Taken together, the understanding of the mechanism underlying the damage caused by IRA and VIS radiations in combination with what is known about UVA and UVB damage should almost certainly provide valuable clues to improve the present strategies for efficient and broad skin photoprotection. Additionally, with the global increase in the incidence of skin cancer and given the knowledge that the development of skin cancer in fair-skinned populations is directly linked to prolonged sun exposure, there is a clear need to revisit the strategies and ingredients used for skin photoprotection. This implies that the photoprotective strategies and ingredients be chosen in a way that they provide adequate protection against broad spectrum UVA, UVB as well as the UVA1/VIS and VIS/IRA spectral regions.

### 2.4. Photoaging Effects of Solar UVA, VIS and IRA

Compared to chronological aging, photoaging is often premature and more pronounced among older individuals who have been regularly exposed to the sun for long periods. Photoaging is characterised by a number of alterations in the skin cells’ components as well as in the ECM. In the dermal compartment, these manifest as disorganised elastin and its microfibrillar component fibrillin, and a consequent depletion of interstitial collagens, the major constituents of the dermal connective tissue [108].

The dual role of solar UVA as an ROS generator and LI enhancer suggests that this radiation is the major contributor to photoaging [5,81]. In addition to alterations to iron homeostasis, the UVA-induced destruction of cellular reducing equivalents (e.g., GSH) and the CAT enzyme also contributes to the further disruption of the redox homeostasis in the skin [12,13,87].

The generation of ROS by UVA irradiation also promotes the synthesis of MMPs via the activation of mitogen-activated protein kinases (MAPKs), such as extracellular signal-regulated kinases (ERKs), c-Jun N-terminal kinase/stress-activated protein kinase (JNK/SAPK), and p38 kinase, to express c-Fos and c-Jun proteins that, in association, form the activator protein 1 (AP-1) transcription factor (see Figure 2). AP-1 has a key role in the transcriptional regulation of MMPs, notably MMP-1, -3 and -9 which are known to transiently increase upon acute exposure to UV radiation, but are also constitutively higher in photodamaged skin [40]. Moreover, AP-1 inhibits the transforming growth factor-β (TGF-β) pathway causing a decrease in procollagen type I production in human skin fibroblasts [109]. In fact, TGF-β cytokine and Smad proteins function as the regulators of the synthesis of TGF-β dependent type I procollagen and collagen. Smad is an acronym from the fusion of *Caenorhabditis elegans Sma* genes and the *Drosophila mad* (Mothers against decapentaplegic) proteins to transduce signals. Smad proteins have been shown to be the main downstream targets of TGF-β receptor kinases, acting with distinct and opposing functions [110]. UVA irradiation is capable of modifying the TGF-β pathway by decreasing the synthesis of TGF-β I and TGF-β II receptors [111]. This in turn will cause the reduced conjugation of TGF-β to the surface of skin fibroblasts, leading to relieved sensitivity of fibroblasts to TGF-β and the downregulation of the phosphorylation of its downstream effector, Smad 2/3. The UVA-mediated decrease in p-Smad 2/3 precludes their translocation to the nucleus and hence their effect on the transcription factors of target genes, including collagen [44,112,113,114] (see Figure 2).

In both keratinocytes and fibroblasts, the expressions of MMP-1 and -3 can also be induced by the tumour necrosis factor (TNF)-α as a result of its upregulation by UVR in both keratinocytes and fibroblasts [115]. In fact, the ROS-mediated induction of MMP-1 is considered as the hallmark of the photoaged skin, leading to collagen breakdown [47,108]. The transcription factor NF-κB can be activated by UVA-induced ROS generation and LI release [78]. Activated NF-κB subunits translocate to the nucleus and induce the upregulation of pro-inflammatory cytokine expression [116]. Furthermore, the activation of NF-κB can induce MMP expression [117].

Overall, UVA radiation plays a crucial role in photoaging in the dermal ECM, by inducing MMPs expression through different pathways. Moreover, the age-related changes occurring in the ECM perturbs the stem cell niche and their self-renewal capacity leading to cell senescence. These effects are also potentiated through the secretion of MMPs [42]. The UVA-induced ROS production has been recognised as the key radiation for these changes, notably via the induction of a number of MMPs and proteases in skin cells and tissue, notably MMP-1 [87]. The extracellular MMP-1 is responsible for the proteolytic degradation of the type-1 and type-3 collagens as well as elastin fibres. The selective activation of MMP-1 (but not TIMP-1) by UVA is considered as a major player in the pathologies associated with photoaging in the human skin [118]. Redox-active LI has also been shown to sensitise the dermal fibroblasts to UVA exposure and to act synergistically with UVA to cause an increase in MMP-1 in iron-loaded and UVA-irradiated fibroblasts [119]. In the same study, UVA concomitantly activated the three members of the MAPK family: ERKs, p38, and JNKs in the dermal fibroblasts. Furthermore, ERKs exhibited an additional activation by iron and contributed to the synergistic increases in MMP-1 in UVA-irradiated fibroblasts. However primary normal human epidermal keratinocytes did not respond to iron or UVA and failed to activate MMP-1, but instead the cellular secretion of TNF-α in the media, stimulated MMP-1 activation in fibroblasts [119].

As mentioned above, the exposure of human skin to either UVA or UVB leads to a selective increase in MMP-1 expression and activity, without inducing its tissue specific inhibitor TIMP-1 [30,120]. This imbalance appears to explain the fate of collagen and elastin fibres that are degraded in the photoaged skin, contributing to increased wrinkle formation and loss of skin elasticity and skin firmness [118]. In addition to MMP-1, UVA also induces MMP-3 and MMP-10 to a certain extent, but these are also activated by UVB. In contrast, MMP-12 induction is specific to UVA radiation. MMP-12 functions as an elastase and is suggested to contribute to elastin degeneration in UVA-induced late solar elastosis [121].

Recent evidence suggests that IRA is also an oxidising component of sunlight as it is able to generate ROS in cells as measured by EPR [86]. In human primary skin fibroblasts, IRA has been shown to affect over 600 genes involved in photoaging and photocarcinogenesis [122]. IRA radiation also contributes to photoaging of the skin (in vitro and in vivo) by activating MMP-1 which is involved in the breakdown of collagen fibres and the formation of coarse wrinkles in the skin [103,122]. Another feature of IRA as a photoaging-inducing agent is related to its ability to reduce type 1 collagen expression in skin cells and to increase the expression of the vascular endothelial growth factor involved in angiogenesis [71].

Irradiation of human skin equivalents with VIS light at a natural exposure level (i.e., equivalent of 20–90 min summer sun in Texas, USA), has also been shown to induce the production of ROS, pro-inflammatory cytokines (i.e., IL-1α, IL-6, GM-CSF, IL-8) and MMP-1 expressions [123]. In an in vivo study, the combination of IRA and VIS radiations also significantly increased the levels of MMP-1 and MMP-9 proteins [124]. Interestingly, commercially available sunscreens had minimal effects on reducing VIS light-induced ROS, suggesting the need for more potent skin photoprotectants against this spectral region of sunlight. In this context, when an AO combination including a Parthenolide-Depleted Feverfew (*Tanacetum parthenium*) extract [125] was added to the UV sunscreen, VIS-generated ROS decreased by 50%. Additionally, only this combined treatment caused a significant decrease in VIS light-induced ROS formation, MMP-1 and inflammatory cytokines’ releases from the epidermal tissue. Taken together, these data strongly suggested that sunscreens containing solely UVA/UVB filters are not effective in protecting the skin against the VIS region of the solar spectrum [123]. Additionally, these findings suggest that other portions of the solar spectrum apart from UV, particularly VIS light, may also contribute to premature skin photoaging. Another in vivo study showed that ROS generated by VIS and IR radiations account for approximately 50% of the total ROS formed by the solar UV waveband. Such a high proportion of ROS production suggests an important contribution from VIS and IR wavebands in potentiating the oxidising effects of solar UV on cell components [76,126]. The question that remains to be addressed at present is whether these wavebands contribute to a similar disruption to iron homeostasis observed with solar UVA radiation.

## 3. Skin, Oxidative Stress and Antioxidant Defense

The term of “oxidative stress” was first used in the 1970s for various deleterious processes. However, later it was defined as an imbalance existing between AO and oxidants in favour of the oxidants, which would potentially lead to deterioration [21]. Oxidative stress occurs when an excessive production of ROS cannot be counteracted by the action of AOs, but also as a perturbation of the cellular redox balance [127]. Therefore, maintaining the skin redox balance between the formation of ROS and their neutralisation is of outmost important, since the organ is constantly exposed to ROS produced from exogenous and endogenous factors. This would prevent the building of oxidative stress, protecting the skin cell constituents and ECM against the deleterious consequences of oxidative damage.

According to the ‘old’ free radical theory of aging [128], free oxygen radicals caused toxicity to the organism, so aging was presumed to be only related to ROS-mediated harmful side attacks on connective tissues and cell components. However, it is now well established that ROS are also produced under normal cellular metabolism. Based on the ‘old’ theory, the overall aging process could slow down if the so-called ‘harmful molecules’ were completely removed from the organs [129]. Among all organs of the body, skin appears to adhere well to this theory. This is because compared to the ROS-load of other organs, skin exhibits a higher ROS load and this affects both intrinsic and extrinsic aging [10]. Significantly, exposomes such as solar UV radiation contribute to up to 80% of skin’s extrinsic photoaging which is distinct from the corporal changes and genetic factors that occur during the intrinsic chronological aging process [130,131]. Nevertheless, free radical theory is not empirical for the skin and hence it was dismissed in 2014. This was because of the complex nature of skin aging and its key dependence on cells’ metabolic organisation, an individual’s genotype and protective systems which were not part of this theory. Currently the theory of skin aging also takes into account the role of genetic and environmental factors, with recognition of cumulative oxidative damage to skin consitutents and ECM from oxidising insults, notably the oxidising components of sunlight such as UVA radiation [21].

Most studies about redox and aging have focused on the static status of oxidative stress levels. This has led to a clear gap in research investigating differential responses to redox challenge during aging. Meng et al. (2017) [132] have proposed a new concept called “Redox-stress Response Capacity (RRC)”, that implies that cells or organisms can generate dynamic redox responses to activate cellular signaling and maintain cellular redox homeostasis. The decay in RRC will, therefore, be a characteristic of aging, which provides a new insight into the redox theory of aging. Under normal conditions, the cellular redox state is tightly regulated by a series of key non-enzymatic and enzymatic AOs to neutralise the excess ROS production, providing a strong AO defense to the skin. These include various AO enzymes, notably SODs, GPxs, thioredoxin reductases (TrxRs) and peroxiredoxins (Prxs) and CAT, all of which play central roles in maintaining the skin’s overall redox balance [133]. The non-enzymatic AO system includes both endogenous and exogenous molecules, notably reduced glutathione (GSH), thioredoxin (Trx), melatonin, A-lipoic acid, carotenoids, polyphenols, vitamins A, C and E, the amino acids methionine and tryptophan, as well as BH4 (6R-L-erythro-5, 6, 7, 8 -tetrahydrobiopterin) and the metalloid selenium [133,134]. In human skin, the AO systems are interdependent, but they collaborate. The treatment with known AOs such as vitamins C (Vit C, ascorbic acid) and E (Vit E, tocopherols), as well as polyphenols are likely to improve the resistance of an organism to ROS-mediated oxidative damage and to prevent skin aging and inflammation [135]. Darker skin phototypes (Fitzpatrick classification) have a higher AO capacity, which implicates the skin pigment melanin as a free radical trap in addition to its absorbing properties [133].

Generally, the redox state of a cell determines its differentiation profile, with an oxidising environment initiating oxidative damage and cell death, and a reducing one promoting proliferation [136]. This is due to the importance of the redox state in signal transduction, enzymatic activation, and DNA/RNA synthesis. Low concentrations of O_2_^•−^ and H_2_O_2_ can stimulate proliferation and enhance survival in a wide variety of cell types. ROS, as redox signaling molecules, can, therefore, play an important physiological role as secondary messengers [137]. In general, ROS are an integral part of the innate immune system and play crucial roles in both the respiratory burst of neutrophils and signal of inflammatory cell chemotaxis into sites of inflammation [138]. Crucially, ROS-generating enzymes are tightly regulated either due to their compartment-specific localisation and/or specific gene expression regulation and AO activity [139]. This is because excess ROS and the closely related reactive nitrogen species (RNS) are capable of disturbing many physiological processes, including cell survival and death. As such, AOs have been shown to prevent apoptosis [140]. In addition, intracellular levels of glutathione represent the major redox buffering system and, therefore, the primary cell cycle mediator [141,142]. Although high ROS/RNS concentrations primarily lead to cell death, low concentrations of these active species can directly affect the activities of a number of transcription factors, notably those of NF-κB, p53, and Nrf2. High levels of ROS/RNS can also affect the regulation of several protein kinase cascades that participate in the regulation of the crosstalk between autophagy, apoptosis, and regeneration [143]. It has been demonstrated that ROS are involved in skin apoptotic processes to remove the aging and abnormal cells. In addition to apoptosis, autophagy can also be activated in a programmed manner in the cellular response to stress or nutrient deprivation [144] to facilitate the degradation of damaged cellular components and to provide the cell with molecular building blocks and energy [145]. Similar to apoptosis, autophagy appears to be involved in skin cell differentiation and tissue regeneration and reconstruction under both physiological and pathological conditions, including wound healing [135]. Additionally, the extent of autophagy decreases with both photoaging and chronological aging of the skin [146].

The concept of the “AO network” relates to the complex intertwined process of redox cycling, where AOs regenerate one another [142,147], such as redox cycles of vitamins E and C. The capacity to regenerate one AO by another is driven by the redox potentials of the [Red/Ox] couple. There is a known link between increased ROS levels and disturbed activities of enzymatic and non-enzymatic AOs in several cellular processes including cancer [148]. Under pathological conditions and exposure to environmental stressors (e.g., pollution, sunlight), the cellular redox state of the skin can be severely altered leading to disturbed AO activity [5,149,150]. During the skin aging, the endogenous AO system is gradually altered, resulting in decreased AO capacity of the elderly skin [32,151].

### 3.1. Superoxide Dismutase (SOD), a Major Antioxidant Enzyme in the Skin

Superoxide dismustases (SODs) intervene in the dismutation of O_2_^•−^ into O_2_ and the less-reactive H_2_O_2_ to protect the cells against oxidative pathologies, as well as premature aging [152,153]. H_2_O_2_ is then reduced into H_2_O via enzymes such as CAT and GPxs [135]. Three isoforms of SODs present in humans include cytosolic Cu/Zn-SOD, mitochondrial Mn-SOD, and extracellular Cu/Zn-SOD.

At the cellular level, the concentrations of O_2_^•−^ has been shown to increase in skin fibroblasts during senescence and skin aging due to higher mitochondrial oxidative stress as a result of an Mn-SOD deficiency [45,154]. Human skin Cu/Zn-SOD resides in the cytoplasm of keratinocytes, where up to 90% of cellular ROS is produced [154]. At the tissue level, the activity of SOD appears to be higher in the epidermis than the dermis in both young and aging skin [155], with the level of Cu/Zn-SOD being higher in the male than the female individuals [156]. In addition, photobiology studies have demonstrated that sun-exposed skin has more SOD expressed than in the non-exposed skin [153,157]. Due to the anti-aging and AO properties of SODs, these enzymes are now used in cosmetics and personal care products.

### 3.2. Catalase, a Major Antioxidant Enzyme in the Skin

Catalase (CAT) is a tetramer of iron-containing heme groups, which effectively decomposes H_2_O_2_ into O_2_ and H_2_O [158]. CAT is largely expressed in the skin, especially in the *Stratum corneum*, with a gradient of activity decreasing towards the surface of the skin [159,160]. During the aging process, the level of CAT activity becomes disproportinate in the skin compartments as it decreases in the dermis and increases in the epidermis of both chronologically aged and photoaged skin. The higher CAT activity in the epidermal compartment of the aged skin is thought to be due to the higher ROS load of the epidermal keratinocytes, whereas the reduction of CAT in the dermis has not yet been elucidated [160,161]. Nevertheless, studies on cultured human cells have demonstrated that as a hemoprotein, CAT can absorb in the UVA spectrum, resulting in its inactivation [162]. Moreover, while acute UV radiation can decrease the activity and the expression of CAT in the irradiated skin, with chronic UV irradiation over a lifetime (approximately 50 years), the CAT activity is increased in the epidermis and dermis of the human skin in vivo. The latter suggests the importance of the CAT enzyme in the skin aging process, and its crucial role in the photoprotection of the skin against UV light [155]. Interestingly, a study that targeted the peroxisomal CAT to the mitochondrial compartment (mCAT) demonstrated significant effects on life span and healthspan extension in mice [163,164].

### 3.3. Glutathione and Thioredoxin Antioxidant Systems in the Skin

Skin cells benefit from two cellular disulphide reductase systems to maintain their intracellular redox balance: the Trx system, comprised of Trx, thioredoxin reductase (TrxR), and NADPH, and the glutathione system, which includes NADPH, glutathione reductase (GR), the reduced glutathione (GSH) and its oxidised form as glutathione disulphide (GSSG) [165]. These systems are also major contributors to the cellular AO defense.

Glutathione is central to the regulation of the cellular redox status. It is considered as one of the most important AO defense as it can either directly interact with ROS, RNS and electrophiles or serve as a cofactor for various enzymes, notably GPxs to protect normal cellular function and cell viability [166]. In the glutathione system, GPxs protect the cells from oxidative damage by reducing H_2_O_2_, thus oxidising GSH to GSSG during the process [167]. At a cellular level, GSH and GSSG act in concert with a number of redox-active molecules, notably NAD(P)H, to regulate and maintain the cellular redox status. In fact, the ratio of GSH/GSSG is a predictable biomarker for cellular redox homeostasis [168]. However, under oxidative stress conditions, the intracellular ratio of GSH and GSSG are lowered which tends to cause increased cytotoxicity due to the increased susceptibility of cells to oxidative damage [87]. GSH is not only involved in thiol redox signaling, but also in cell proliferation and differentiation and in the regulation of cell death, including apoptotic pathways. Lowered GSH metabolism and a low GSH/GSSG ratio following oxidative stress are associated with mitochondrial dysfunctions and constitute a critical factor in several pathologies [169,170,171].

Due to its central role in the regulation of the cellular redox status, GSH is also present in the intracellular compartments/organelles in a greatly reduced state and possesses specific vital functions therein. In fact, both the integrity of cell and subcellular membranes are highly dependent on the presence of GSH and GPx [172]. During aging, GSH levels tend to decrease in some organelles and tissues which make the cells susceptible to oxidative damage. In the cytoplasm, GSSG is usually in the order of at least 1% of the total cellular glutathione content. In the nucleus, GSH maintains the redox status of the sulfhydryl groups of the proteins required for the biosynthesis of nucleic acids. Similarly, GSH tends to reduce ribonucleotides to yield deoxyribonucleotides by ribonucleotide reductase [173]. In the endoplasmic reticulum (ER), glutathione is oxidised with a [GSH]/[GSSG] ratio of ca 3:1 which appears to be essential for the oxidative folding of the nascent proteins of the organelle.

Mitochondrial GSH (mGSH) concentration is around 10–15% of total cellular GSH, which is considered high given the small volume of these vital organelles when compared to cytosol that contains about 90% of cellular GSH. The higher mGSH has been linked to both the higher survival of cells against apoptotic cell death and mitochondrial ROS production [174]. The concerted action of high ROS production and calcium as well as a high mitochondrial labile iron pool (mLIP) observed in some pathologies or under conditions of oxidative stress, including exposure to environmental oxidising agents, notably the UVA component of sunlight, can trigger the mechanism of cell death via apoptosis or necrosis, depending on the extent of the oxidative insult [25,175]. The study of subcellular localisation of the glutathione system in aging fibroblasts showed that GSH was localised in all subcellular compartments but GPx and GR activities were only restricted to cytoplasm and mitochondrial compartments, respectively. Furthermore, the GSH concentration as well as the activities of GPx and GR are significantly modulated in aged fibroblasts, suggesting that the glutathione system may play a role in cell degeneration associated with aging [176].

Due to its anti-melanogenic properties, GSH has recently gained popularity for its systemic use for skin-lightening [177,178]. There are three intertwined mechanisms that contribute to the anti-melanogenic properties of GSH. These include its inhibitory effect on tyrosinase enzyme, its ability to modify the melanogenesis process from the darker eumelanin to the lighter phaeomelanin, and its strong free radical scavenging properties [179]. Due to limited absorption and bioavailability of glutathione in oral form [180], topical application strategies have expanded, notably the use of topical GSSG for skin whitening and improving skin conditions and that of a GSH-loaded dissolving microneedle patch prepared with hyaluronic acid [181,182], to improve skin permeability, while reducing the unpleasant odor of GSH.

In humans, GPxs constitute eight isoforms, called GPx1 to GPx8, which catalyse the breakdown of H_2_O_2_ and related hydroperoxides to prevent a Fenton reaction and subsequent lipid peroxidation [183]. GPx1-4 and GPx6 that contain a selenocysteine residue (SeCys) in their structure use GSH as the reducing equivalent to catalyse the reduction of H_2_O_2_ and lipid peroxides, whereas those non-selenium containing GPxs congeners, called TGPx, do reduce ROS using Trx, which functions as an ROS sensor in multiple cellular pathways, including signal transduction [184,185,186,187,188]. The cytosolic GPx4 is also a known regulator of the iron-related death called ‘ferroptosis’, as it can reduce the hydroperoxy groups of complex lipids and silence the lipoxygenases [188]. GPx1 is also capable of preventing peroxide-mediated oxidative damage, lipid peroxidation, and protein degradation. Increased activity of GPx1 can also inhibit H_2_O_2_-induced apoptotic and the related LI-catalysed necrotic cell death [186,187,188,189]. Selenium consumption has also been linked with a decrease in GPx ctiveity, as observed in rat plasma in extreme old age, and this may be used as an indicator of physiological aging [190].

The ROS-sensor Trx is typically a small (12kDa) reductase, catalysing protein disulphide/dithiol change with a conserved-CGPC-active site motif [191]. As a strong redox-regulating AO protein, Trx is capable of quenching ROS under oxidative stress conditions as well as attenuating the age-related deterioration during senescence, by interfering with the cellular redox state, and thereby extending the life span of the organism. The protective role of Trx against UVB-induced skin injury and peroxidative damage is well-established [133]. The Trx system has also been suggested as an index marker for cellular proliferation and senescence due to its correlative expression with cellular conditions [192]. The AO functions of Trx also include cellular reductive reactions for vital enzymes such as ribonucleotide reductase involved in DNA synthesis, peroxiredoxin (Prx) that reduces peroxides [193], and methionine sulphoxide reductases (Msrs) that repair the free and protein-bound S- and R-methionine sulphoxides back to methionine [194,195]. Trx also regulates the activities of a significant number of redox-sensitive transcription factors, notably NF-κB, Nrf2 and P53 [196,197]. In addition, Trx plays an important role in maintaining a reduced environment in the cells through thiol-disulphide exchange reactions, thereby acting as a protective agent against oxidative stress in cells and tissues [191]. In the skin, the anti-inflammatory action of Trx relates to its ability to inhibit the local formation of inflammatory cytokines and chemokines, notably TNF-α, Il-1β, IL-6, cytokine-induced neutrophil chemoattractant ligand 1 (CXCL-1), and monocyte chemoattractant protein-1 (MCP-1) [198].

Topical application of Trx has been considered as a promising new approach for the therapy of various anti-inflammatory skin disorders (e.g., [199]). There are so far three identified isoforms of human Trx with the cytosolic Trx1 exhibiting anti-senescent properties in the skin fibroblasts [200,201,202,203]. The cytosolic TrxR1 and the mitochondrial TrxR2 enzymes that govern the activities of cytosolic Trx1 and mitochondrial Trx2 have been shown to contribute to cell proliferation and apoptosis regulation, respectively [204]. Recently, an association between mitochondrial TrxR2/Trx2 levels/activity and lifespan in several independent in vivo models has been described that may be the result of either improved mitochondrial AO defense and/or changes in sensitivity to apoptotic signaling [205].

Homodimeric flavoprotein TrxR is a member of the pyridine nucleotide-disulphide oxidoreductase family, which also includes GR [206,207]. TrxR is responsible for the NADPH-mediated reduction of oxidised Trx. The reduced Trx can then donate electrons to thioredoxin peroxidase (TrxPx) to reduce H_2_O_2_ to H_2_O. In this context, the level of membrane associated TrxR correlates with the skin types I–VI (Fitzpatrick classification), with darker skin having a higher enzyme activity than very fair skin, contributing to effective AO defense in this organ. Additionally, the UVB-mediated generation of H_2_O_2_ in the epidermis has been shown to be accompanied with an upregulation of TrxR mRNA. However, sustained exposure to high levels of H_2_O_2_ causes the downregulation of cytosolic TrxR1 as a result of p53 induction. These findings are in line with the notion that H_2_O_2_ levels can control the extent of TrxR activities. A new function for the Trx system in epidermal cells involves the control of (6R)-L-erythro 5,6,7,8 tetrahydrobiopterin (6BH4) homeostasis. Epidermal melanocytes have been shown to be lethally affected by H_2_O_2_-mediated oxidation of 6BH4 to yield 6-biopterin, as observed in vivo in vitiligo [208].

A possible approach to attack ROS-mediated disorders for both preventive and treatment means is based on targeting a cytoprotective signaling pathway, the Kelch-like Ech-associated protein 1 (Keap1)–Nrf2 pathway which, in addition to other activities, regulates the AO response and, therefore, maintains the skin redox balance as detailed below.

### 3.4. Nuclear Factor E2-Related Factor 2 in Skin Redox Homeostasis

The redox-sensitive transcription factor Nrf2 is recognised as one of the key players in skin homeostasis and renovation, as well as in many skin disorders [209]. Nrf2 is expressed in all skin cell types and acts as a regulator of oxidative stress to protect skin cells against the oxidative damage and the resulting cellular dysfunction exerted by oxidising agents such as H_2_O_2_ and UVA [210,211]. In UVA-irradiated skin cells, GSH depletion [212] directly influences the intracellular redox homeostasis, which in turn activates the redox-sensitive Nrf2 [83]. In this context, Nrf2 has been implicated in the protection of skin fibroblasts against UVA-induced lipid peroxidation, inflammation and MMPs expressions [213].

Nrf2 protein is composed of two subunits (p45 and Maf) which, through interaction with DNA, regulate the expression of a considerable number of genes. Under physiological conditions due to cellular balance between ROS and activity of AOs, Nrf2 remains in a suppressed form in the cytoplasm. This suppression occurs adjacent to the cellular cytoskeleton by the interaction of Nrf2 with Keap1 and Cullin 3 (Cul3), a protein of the E3 ligase family [214,215]. Keap1 belongs to the BTB-Kelch family of proteins [216] and serves both as a stress sensor and an adaptor component for Cul3-based ubiquitin E3 ligase [217]. Cul3 is related to Keap1’s function as the regulator of the degradation of Nrf2 by forming a complex with ring box 1 (RBX1) to yield a functional E3 ubiquitin ligase. In the Keap1-Cul3-RBX1 E3 ubiquitin ligase complex, Keap1 is the substrate adaptor while RBX1 binds to the ubiquitin loaded E2-ubiquitin conjugating enzyme, and Cul3 forms the scaffold to join Keap-1 and RBX1 together. This complex functions to correctly orientate the Nrf2-bound Keap-1 and the E2-bound RBX1 to facilitate ubiquitination of Nrf2 and its proteasome-dependent degradation [218,219]. Under physiological conditions with an appropriate balance of ROS and AOs, Keap1 has been shown to constitutively ubiquitinate Nrf2 and cause the rapid proteasomal degradation of Nrf2 to maintain the basal level of cellular Nrf2 along with its target gene expressions. Under conditions of oxidative stress, electrophiles and ROS act to modify the reactive cysteine residues of Keap1 which will then inhibit its activity as an E3 ligase component. This leads to the stabilisation of Nrf2 and its translocation to the nucleus, where it forms a heterodimer with other transcription factors, such as small Maf, which in turn binds to the 5′-upstream Cis-acting regulatory sequence, termed AO response elements (ARE) or electrophile response elements (EpRE), located in the promoter region of genes encoding various AOs and phase II detoxifying enzymes, [220] and thereby promotes the transcription of over 250 genes. These genes encode distinct homeostatic functions involved in redox metabolism, the regulation of inflammation, and proteostasis [221], including NAD(*P*)H:quinone oxidoreductase 1 (NQO1) [222], glutathione S-transferase (GST) [223], GR [224], SOD, CAT, glutathione S-transferase (GST), TrxR1 and GPx2 [225,226,227,228].

Short-term activation of the Nrf2 signaling pathway appears, therefore, to be important in protection against oxidative damage to the skin constituents, notably against both UVA- and UVB-induced cutaneous cell apoptosis [229,230,231]. In this context, Nrf2 is able to regulate a range of anti-apoptotic molecules involved in the cellular response to oxidative stress including UV components of sunlight, especially in the outer layers of the skin [232]. Additionally, Nrf2 expression and activity appears to follow a gradient in the murine epidermis with the more differentiated, supra-basal cells expressing much higher Nrf2 levels (including its target genes) than the undifferentiated basal cells [233,234]. The differentiation of human keratinocytes has been linked to Nrf2-mediated NQO1 induction [235]. In a series of experiments involving human keratinocytes, UVA exerted a positive effect on Nrf2 activity and its target genes versus solar light (a mixture of 16.8 J/cm^2^ UVA and 480 mJ/cm^2^ UVB) [236], suggesting that UVB is a suppressor of Nrf2 activity and its target genes expression. The latter was confirmed in a study using cultured human keratinocytes and melanocytes with UVB, showing the downregulation of Nrf2 and its target genes which was suppressible with the α-melanocyte-stimulating hormone (α-MSH). This highlighted the cytoprotective and AO potential of α-MSH and possible related melanocortin peptides [237]. Other recent studies have highlighted the importance of autophagy in regulating physiological skin colour [238,239,240].

Nrf2 also participates in the clearance of any oxidised/damaged cell organelles and proteins upon cellular redox alterations, notably following UVA irradiation of skin keratinocytes [241,242]. Autophagy has also been shown to be crucial for the limitation of Nrf2 activation in keratinocytes, in particular in response to UVA irradiation [243,244]. A recent study has also demonstrated the anti-melanogenic mechanisms of ellagic acid, a natural phenol compound, through autophagy induction in a cultured murine melanocyte cell line, as well as the suppression of UVA-mediated activation of α-MSH pathways via Nrf2 activation in human keratinocytes. Interestingly, pterostilbene, a natural polyphenol, inhibited the UVA-induced and ROS-mediated α-MSH production in human keratinocytes via Nrf2-mediated HO-1 and γ-Glutamate-cysteine ligase catalytic subunit (γ-GCLC) activation [240].

In the skin, the Broad complex, Tramtrack, Bric-a Bric (BTB) and Cap ’n’ collar (CNC) homology (Bach) family members Bach1 and Bach2 are considered as key players in UV-induced oxidative damage. UVA radiation has been shown to modulate the cellular redox state by Bach1 [83]. The ARE is normally suppressed by a heterodimer formed by the sMaf and Bach-1 proteins, preventing Nrf2 heterodimerisation and binding to the ARE [245,246]. The response of Bach1-deficient mouse embryonic fibroblasts to oxidative stress is very rapid and Bach1 can suppress cellular senescence by recruiting and binding to p53 to inhibit the activation of target genes [247]. On the other hand, Bach2 is also sensitive to UV-mediated oxidative damage and has recently been shown to suppress the UVA-mediated cell senescence via autophagy in skin fibroblasts. It is, therefore, suggested that Bach2 can be a potential target for the therapy of UV-induced photoaging [248].

In addition to UV radiation, a number of natural and synthetic compounds that induce ARE genes via Nrf2 have been identified. These include phytochemicals and derivatives such as CDDO and sulforaphane, therapeutics such as oltipraz and auranofin, environmental agents such as paraquat and arsenic, and endogenous chemicals such as NO, 15d-PGJ_2_, nitro-fatty acids, and 4-hydroxynonenal (4-HNE)] [249,250,251]. Inducers are structurally diverse and have few common properties, except for their ability to modify -SH at rates closely correlating with their potency for induction of NQO1.

## 4. Natural-Based Antioxidants for Skin Protection

Recognition of the crucial role of exposomes notably, UVA, VIS and IRA as oxidising components of sunlight to skin photodamage and premature photoaging, suggest photoprotection by AOs. In the skin photoprotection field, AOs have been shown to enhance the endogenous AO capacity of the skin and to promote the neutralisation of ROS generated by the solar radiation components. While cells and skin tissue possess a variety of AO enzymes and molecules, recent trends towards the use of natural products have advocated that plants provide a rich source of natural photoprotective AOs with strong anti-aging properties. These include several classes of botanical-, fungal- and marine-based compounds, notably polyphenols, monoterpenes, flavonoids, carotenoids, organosulphides, indoles, chromanols and chromenols. These compounds have been shown to stimulate immune and anti-inflammatory responses, modulate AOs, detoxify the cells and tissues, alter gene expression, and thereby restore the redox homeostasis and protect the skin against the features of both intrinsic chronological aging and extrinsic photoaging [252,253,254].

Additionally, a series of natural compounds have been identified for their effective photoprotective /UV-absorbing properties, providing new options and choices for sunscreen formulations. These include propolis cinnamic acids, tea polyphenols, grape seed proanthocyanidins, milk thistle silymarins, algae MAAs, algae terpenes, lichen polyphenols, lignin, and melanin (reviewed in [255,256,257]). A recent study has also demonstrated a positive correlation between the altitude at which the sun-protective plants grow up and the bio-production of metabolites with both AO and photoprotective properties [258]. Therefore, boosting the AO capacity of skin cells by using exogenous AOs appears to be a valuable strategy for preventing UV-induced skin photodamage and photoaging.

In view of the concomitant and inter-related alterations of intracellular iron- and redox-homeostasis that occur upon oxidative stress conditions, such as the exposure of skin cells to oxidising components of sunlight, and lead to premature skin photoaging, it is necessary to revisit the selection criteria for natural AOs to include effective redox-balancing and/or iron-chelating properties, especially when devising skin care products and sunscreen formulations. More specifically, the dual role of UVA as ROS generator and LI enhancer and its established roles in photoaging and photocarcinogenesis suggest skin photoprotection by natural AOs with potent iron chelating properties. This notion is strengthened by a number of studies demonstrating that in addition to ROS neutralisation, the concomitant chelation of excess LI release is necessary to obtain an effective photoprotection in UVA-irradiated cells, while also restoring the disrupted cellular iron- and redox-homeostasis [13,16,24,259,260,261]. In this context, several polyphenols and flavonoid compounds may fulfil the desired properties as both potent AOs and iron chelators. Alternatively, natural-based AOs that are capable of inducing the key redox Nrf2 transcription factor may be powerful enough *per se* to reinstate the redox homeostasis of the oxidatively-compromised skin cells by promoting the transcripion and translation of a considerable number of AO enzymes and pathways. Lastly a number of additional natural AO types emerge that not only possess strong ROS quenching/scavenging/neutralisation properties, but also exhibit strong anti-inflammatory and anti-cancer properties that are independent of the Nrf2 pathway. The potential of some of these AO categories as anti-aging and redox-balancing compounds for skincare and sunscreen formulations are discussed below.

### 4.1. Chromanols and Chromenols as Promising Skin Antiaging and Photoprotectants

Chromanols and chromenols are collective terms for about 230 structures derived from photosynthetic organisms such as plants, algae, cyanobacteria, fungi, corals, sponges, and tunicates [258]. Vitamin E (vit E) represents the most widely distributed and abundant chromanol in nature. The term vit E comprises different lipophilic molecules that consist of the chromanol ring structure with a covalently bound phytyl-like sidechain. Since vit E is essential for mammals, including humans, they all rely on these organisms for a supply of this lipid-soluble factor. The structural diversity of chromanols appears to be related to their side chain modifications and biological activity [258]. Photosynthetic organisms are particularly susceptible to oxidative damage because oxygenic photosynthesis causes an increased oxygen concentration in chloroplasts. Chromanols are also important prenyllipid AO constituents which are capable of scavenging oxygen and organic radicals, as well as quenching and scavenging ^1^O_2_, and thereby protecting them against ROS and consequent structural damages [262]. In fact, for many years, vit E was recognised solely as an AO due to its ^1^O_2_ quenching property. For example, in UVA-irradiated skin cells, vit E supplementation blocked the chain of lipid peroxidation in the cell membranes and protected the cells against oxidative cell death by virtue of its ^1^O_2_ quenching and scavenging abilities [5,16].

In view of their established photoprotective and antiaging properties, vit E and its derivatives remain strong candidates as ingredients for sunscreen formulations and skin care products [263,264,265]. Furthermore, the oxidative modifications of the terminal side-chain of tocopherols have also been shown to increase the anti-inflammatory activities of these compounds, indicating that both vit E and its metabolites possess additional non-AO physiological activities in cells and the body. Examples include vit E’s modulatory roles in gene expression and enzyme activities, as well as its interference with signaling cascades. Some of the regulatory effects also include the suppression of inflammatory mediators, ROS and adhesion molecules, the induction of scavenger receptors, as well as the activation of NF-κB. Sargachromanols, sargachromenols, and amplexichromanols that possess tocotrienol-derived backbones are expected to have similar biological activities [266].

Dehydro-δ-tocotrienol is the potential biosynthetic precursor for most of the chromenols found in brown algae. It is known as sargachromenol and was originally isolated from *Sargassum tortile*, collected at the Japanese Tanabe Bay. A lipid extract of the algae exhibited high cytotoxic activity and was used as a skin lightening agent. Sargachromenol gained attention on the skin care and skin health research world since it was reported that it has strong anti-inflammatory activity and anti-hyperproliferative properties in the skin cells. For example, sargachromenol treatment was able to induce apoptosis in hyperproliferative human HaCaT keratinocytes and to protect the skin fibroblasts against UVA-induced damage by suppressing the activity of several MMPs, notably MMP-1, -2 and -9 [267,268]. Sargachromenol also received attention in drug research since it exhibited inhibitory activity against enzymes related to Alzheimer’s disease. Furthermore, it demonstrated both strong anti-inflammatory activity and antiproliferative properties in skin cells [258]. Sargachromenols (mostly δ-SCE) have been identified as potent anti-inflammatory compounds based on their inhibitory effect on NO^•^ production in lipopolysaccharide-treated immortalised murine microglial BV-2 cells. Moreover, these compounds exhibit potent anti-photoaging and anti-cholinesterase activities [266].

Six meroterpenoids of chromene class were also isolated from the brown algae *Sargassum siliquastrum*. These compounds possess strong AO activity as they were capable of effectively inhibiting the intracellular ROS formation and lipid peroxidation induced by H_2_O_2_ and increasing the intracellular GSH level in human fibrosarcoma cells [269]. *S. siliquastrum* also contains other promising AO families, notably fucoxanthin which is a carotenoid with strong AO activity, as well as anticancer, antidiabetic, anti-inflammatory, UVB-photoprotective and anti-photoaging properties [270,271]. Fucoxanthin incorporated in a cream formulation was also found to prevent UVB-induced acute erythema when topically applied on hairless mice. This anti-inflammatory response was mediated by the downregulation of inducible NOS (iNOS) and cyclooxygenase-2 (COX-2) proteins and the upregulation of HO-1 through Nrf-2 pathways and, hence, its redox homeostasis boosting properties [272]. There are also a number of other components isolated from the brown algae *S. sagamianum*, including plastoquinones, sargaquinoic acid, and sargachromenol, all of which exhibit photoprotection against UVB and also have potential as anti-photoaging compounds [267].

As mentioned previously, the causal relationships between prolonged UVB or UVA radiations exposures and skin sunburns, photoaging, and cancer are well-established. However, in view of the recent studies demonstrating the need for broad spectrum solar phoprotection spanning from UVB to IR as well as for environmental insults, notably pollution, the design of skin care products is changing gradually [71]. In this context, recent skin formulas include the innovative new ingredient dimethylmethoxy chromanol (DMC, 3,4dihydro-6-hydroxy-2,2-dimethyl-7-methoxy-1(2H)benzopyran), which captures both nitrogen and oxygen radicals to limit oxidative stress, providing protection against ROS damage induced by environmental pollution and other sources such as solar oxidising radiations [273]. To test the hypothesis that the incorporation of AOs into sunscreens provides additional skin photoprotection against harmful ROS production by terrestrial sunlight radiations, DMC and *Spirulina* were added into broad-spectrum sunscreens and tested in a 3-month, single-blind clinical study with 44 healthy subjects. The results confirmed the potent AO properties of theses ingredients in the sunscreen formulation as both skin pigmentation and dermal collagen degradation were significantly decreased. This was accompanied by a significant improvement in the skin’s net elasticity after 84 days of treatment, when compared to the control group that were treated with the sunscreen formulation alone [274]. In another study, the ^1^O_2_ quenching ability of DMC was also evaluated to confirm its AO potency in cosmetic and pharmaceutical formulations. As highlighted in this review, ^1^O_2_ is a non-radical ROS that is believed to play a major role in many photooxidation processes in connection with diverse photobiological processes, such as skin photoaging and photocarcinogenesis. Treatment of ex vivo porcine skin samples with DMC showed a clear reduction in the ^1^O_2_ lifetime and emission intensity when compared to untreated samples [275]. In addition, a number of studies demonstrated the stronger AO properties of DMC when compared to grape seed or tea extracts. Topical application of DMC cream containing the active ingredient to the skin of volunteers, demonstrated a significant rise in the AO capacity in their skin by 21.3% and 36.7% after 14 and 28 days, respectively. Taken together, studies on DMC demonstrate its suitability to be incorporated into cosmetic formulations to prevent premature skin aging due to environmental aggressors [276].

Chromanols are not only present in algae, but also in diverse forms in plants. A recent study discovered two new dilignans with a 2-phenyl-3-chromanol motif from the stem barks of *Magnolia obovata*, commonly called Japanese bigleaf magnolia, white bark magnolia or white leaf magnolia [277].

### 4.2. Polyphenols as Skin Antiaging and Photoprotecive Agents

Natural-based products belonging to the family of polyphenols encompass a large variety of vegetables, fruits, as well as nuts, seeds, bark, and flowers. This family provides an important source of dietary AOs, anti-inflammatory and potentially anticancer products [278]. Some of the key members of this family include flavonoids, phenolic acids, and stilbenes. Examples of flavonoids include flavonols, proanthocyanidins, anthocyanins, catechins and flavovones. Phenolic acids include benzoic, gallic and cinnamic acids. Stilbenes derived from plants include tea, grape, bergamot, fernblock, rooibos, grapefruit, and red orange [279,280].

Polyphenols have been extensively studied in cultured skin cells as well as in skin reconstructs and human skin, and their beneficial properties are well known in the field of skin photoprotection and photo-(chemo)-prevention [257,281,282,283].

The potent AO activities of flavonoids and other polyphenols have been established in vitro, especially their ability to scavenge a wide range of ROS, including ^•^OH radicals, peroxyl radicals, hypochlorous acid and occasionally O_2_^•−^ radicals [284]. Many flavonoids chelate transition metal ions, such as iron and copper, decreasing their ability to promote reactive species formation [285]. It is well known that catechol and gallol and the many functionalised derivatives thereof (including most polyphenol compounds) are effective metal chelators [286]. In this context, we have previously demonstrated that physiologically relevant concentrations of either (−)-epicatechin (EC) or methylated EC (3′-O-methyl epicatechin, MeOEC) which is its major human metabolite, significantly prevented the UVA-mediated release of LI by protecting the lysosomal organelles against radiation-induced damage [287]. Many of these bioactive natural compounds are capable of regulating both iron metabolism and redox state, presumably through interactive mechanisms. For example, (−)-epigallocatechin-3-gallate (EGCG) is a potent iron chelator, but also an Nrf2 inducer and anthocyanins, in addition to having an iron chelating ability, are also able to increase the level of AO enzymes, notably GPx4, SOD and the total AO capacity mediated by iron regulatory hormone hepcidin and iron export protein ferroportin [288].

The specific sets of pro-inflammatory mediators that are secreted by senescent cells and are responsible for significant changes in the structure and function of tissues are collectively called SASPs (senescence-associated secretory phenotype). Compounds that have the ability to selectively eliminate the senescent cells or inhibit SASP are being recognised as anti-senescent (anti-aging) compounds in chronological skin aging. In this context, flavonoids and their metabolites show promising anti-aging features by targeting key cellular pathways involved in the regulation of cellular senescence and SASP [289]. In addition to their AO properties, flavonoids and other phenols as complex molecules possess multiple potential actions, notably they can act to inhibit the activities of a variety of enzymes, thereby affecting cellular signal transduction pathways and interacting with sirtuins. Examples of enzymes include MMPs, telomerases, COXs, angiotensin-converting enzymes, lipoxygenases, sulphotransferases, glutamate dehydrogenase, xanthine oxidase, proteasomes, and cytochrome P450 enzymes [284].

Below, two selected flavonoids are discussed in the context of their multiple properties as potent AOs, anti-inflammatory and anti-cancer agents. Their strong redox- and/or iron-homeostasis balancing properties against skin aging, photoaging and photocarcinogenesis are also highlighted in view of recent studies in the field of skin antiaging, photoprotection and a number of skin-related oxidative conditions.

#### 4.2.1. Apigenin

Apigenin, a water-insoluble yellow crystalline powder, is a common flavonoid found as a single ingredient in chamomile tea, obtained from the dried flowers of *Matricaria chamomilla*. Apigenin is abundant in a variety of natural sources, including fruits and vegetables [290]. The flavone C-glycosides derived from apigenin is one of the main compounds found in *Cosmos caudatus* extract. In vitro tests using *C. caudatus* leaf water extract demonstrated that in addition to its AO activity, it also exhibits anti-collagenase, anti-elastase, and anti-tyrosinase activity, which makes it suitable as an antiaging ingredient in skin care products [291]. *Clerodendrum petasites*, a shrub with white flowers, is one of the plants that contain apigenin as a dominant compound. It is widely used in Thai traditional medicine for inflammation, skin disorders and other conditions. *C. petasites* has a positive therapeutic effect on inflammation, probably via prostaglandin inhibition [292].

Different studies have assessed the effects of apigenin treatment on random skin flap survival. Dorsal skin flaps were transplanted into rats, which received apigenin in different concentrations, or in combination with other compounds. Animals that received a high dose of apigenin showed a higher average flap survival area, microcirculatory flow, increased SOD activity, and higher vascular endothelial growth factor expression levels compared with the other two groups. Furthermore, the levels of malondialdehyde (MDA, a lipid peroxidation biomarker) and pro-inflammatory cytokines were significantly decreased in this group, suggesting the potential usefulness of apigenin in preventing skin flap tissue necrosis. Apigenin also promoted angiogenesis, inhibited cell apoptosis, and lowered oxidative stress by mediating autophagy, thus improving the survival rate of random skin flaps. These results confirmed apigenin’s potent AO, angiogenic, and anti-inflammatory properties in the skin [293,294].

As mentioned before, the transcription factor Nrf2 is a crucial regulator for the maintenance of oxidative stress. A perilesional melanocyte cell line from vitiligo treated with apigenin exhibited enhanced viability and expression of the cellular AO enzymes SOD, CAT, and GPx, but inhibited production of MDA. Additionally, apigenin treatment influenced Nrf2 expression and its nuclear localisation, which was significantly increased [295].

The effect of apigenin on murine macrophage, rat basophilic leukaemia, and human immortalised keratinocyte cells were analysed regarding the apigenin-mediated amelioration of skin disease and its applicability as a functional ingredient. Apigenin significantly inhibited NO^•^ production, inflammation-inducible cytokines expression (IL-1, IL-6, IL-4, IL-5), COX-2 and inducible NOS and TNF-α. On the other hand, apigenin significantly induced the expression of filaggrin, loricrin, aquaporin-3, hyaluronic acid, and hyaluronic acid synthase-1, -2, and -3, which are considered as the main components of the physical barrier of the skin. It also promoted the expression of human β-defensin 1, -2, and -3, and cathelicidin- antimicrobial peptides known to play an important role in the skin as chemical barriers. This study demonstrated that besides improving the functions of the physical and chemical skin barriers, apigenin could also alleviate psoriasis, acne, and atopic dermatitis [296].

The antiaging and AO properties of apigenin were also observed with extracts of *Humulus japonicus*, containing apigenin-8-C-glucoside, which showed a strong inhibitory effect on the MMP-1 secretion on UVB-irradiated human skin fibroblasts. Additionally, apigenin treatment of skin fibroblasts strongly promoted the elevation of AO-related proteins, Nrf2, and HO-1 in a dose- and time-dependent manner. The study further revealed that the extract suppressed the UVB-induced MMP-1 production by inhibiting the phosphorylation of the MAPKs and AP-1 [297]. Moreover, apigenin has the ability to restore the proper function of the skin (e.g., DNA repair and cell viability) in both UVA- and UVB-irradiated human keratinocytes and dermal fibroblasts [298,299]. This is due to apigenin’s anti-inflammatory ability to inhibit the UVA- and UVB-mediated expression of COX-2 and the NF-κB pathway [300]. Apigenin’s interaction with the NF-κB pathway in the bleomycin-induced senescent fibroblasts appears to be responsible for the decreased secretion of SASP factors from the cells, notably IL-6 and IL-8 [301]. Interestingly, apigenin inhibits the production of interferon-γ-inducible protein 10 (IP10, CXCL10), a component of SASP secreted by senescent fibroblasts that can elicit an abnormal immune response in the elderly [289].

Exposure to UV radiation elicits melanogenesis and pigmentation in the skin. Apigenin-treated keratinocytes isolated for the epidermis of mouse skin specimens were exposed to UV radiation. Chronic UVB-induced cutaneous pigmentation, macrophage migration inhibitory factor (MIF) expression, and the related factors induced by MIF were inhibited by apigenin. Taken together, the study suggested that apigenin regulates UVB-induced hyperpigmentation through its AO properties as well as through its ability to inhibit the casein kinase 2-mediated expression of MIF [302].

Apigenin has strong photoprotective properties against UVA and UVB radiation-induced destruction of the dermal collagen matrix, and the subsequent loss of elasticity and skin dryness, by decreasing the activity of MMP-1. The induction of collagen type I and III de novo synthesis has been observed both in vitro in apigenin-treated dermal fibroblasts, and in vivo in mice where apigenin provoked an increase in dermal thickness and collagen deposition in the dermis of the animals [303]. Subsequently, topical apigenin clinical trials further confirmed its anti-aging effects by exhibiting marked improvements in biomarkers of skin aging, notably skin firmness, elasticity, fine wrinkling as well as adequate skin hydration [298,304].

Dietary intake of flavonoid-rich products has shown to ensure DNA protection of skin cells exposed to carcinogenic factors, such as UV radiation. Among flavonoid compounds, apigenin, quercetin, silymarin, diosmetin, genistein, fisetin, and luteolin, have been identified as potential anti-cancer agents in skin cancers. Hydrogels consisting of gellan gum and chitosan crosslinked with poly (ethylene glycol) loaded with apigenin were developed as a wound dressing for skin cancer. The results on rat wound models showed a 96.11% release of the bioactive compound within 24 h and significant AO activity. While the unique properties of the hydrogels in terms of biocompatibility, biodegradability, moisture, and AO efficiency are considerably promising for wound healing and regeneration, the release of the bioactive compound should be gradual in order to ensure an optimal concentration at the wound site for longer periods [305].

Despite all the remarkable effects of apigenin, its poor water solubility and low storage stability have limited its application feasibility on the pharmaceutical field. To address this issue, a study developed a better topical delivery system where apigenin was efficiently incorporated into nanoemulsions. Consequently, the chemical stability and AO ability of apigenin as well as its skin deposition was significantly improved by the nanoemulsion-based formulation [306].

#### 4.2.2. Baicalein and Baicalin

Baicalein and its glycoside, baicalin, constitute the major bioactive compounds found in *Scutellaria baicalensis* (i.e., Chinese skullcap). These falavonoids possess strong AO and iron-chelating properties capable of inhibiting the LI-promoted Fenton chemistry via a range of mechanisms in cancer, bacterial infections, sunlight exposure and oxidative stress diseases [307,308,309,310,311,312]. Their properties also encompass antipyretic, analgesic, anti-inflammatory, antiallergic, antimicrobial, anti-senescence/-photoaging, immunomodulatory, antimutagenic, anticancer and antitumour effects [313,314,315,316,317]. Baicalein also exhibits remarkable anti-ferroptotic activity in normal and cancer cells, with inhibitory effects comparable to that of desferrioxamine mesylate, a clinical iron chelator and a known inhibitor of ferroptosis [318,319]. Ferroptosis is an LI-mediated and lipid peroxidation-driven mode of cell death that can be induced under redox imbalance and oxidative stress conditions [320,321]. Accordingly, the inhibitory effect of baicalein on ferroptosis appears to be related to its dual iron-chelating and AO properties as it concomitantly limits LI accumulation, GSH depletion, lipid peroxidation and GPx4 degradation induced by oxidising agents that induce ferroptosis [318]. These properties of baicalein will, therefore, be beneficial to UVA-irradiated skin cells as a potent photoprotective agent, as the UVA component of sunlight promotes LI accumulation and lipid peroxidation, both of which can be abolished by iron chelator pre-treatment, such as desferrioxamine mesylate. In this context, a recent study has demonstrated strong UVA absorbing and antiaging properties of balcalein against UVA irradiation with a signficant decrease in the level of UVA-induced lipid peroxidation byproduct, MDA [322]. Interestingly, the activation of the Keapl-Nrf2 pathway has been shown to protect the cells against ferroptosis [323,324]. In this cotext, both baicalein and baicalin are potent inducers of Nrf2 AO pathways in a variety of conditions, from drug-induced toxicity to redox imbalance and related oxidative stress diseases [325]. For example, in human vitiligo melanocytes, baicalein has shown a protective effect against H_2_O_2_-induced oxidative stress through the activation of the Nrf2 signaling pathway [326].

In the skin, the *S. baicalensis* extract has been shown to neutralise ROS, decrease the levels of oxidative stress and exert anti-aging properties [327,328,329]. In vivo, baicalin has demonstrated potent photoprotective properties against UVB-induced DNA photodamage (CPDs) and epidermal hyperplasia in the skin [330]. The latter effects of baicalin have been linked to its strong AO and anti-inflammatory properties due to its modulatory effects on NF-κB, COX-1, and iNOS activities [331,332]. In both in vitro and in vivo models of skin photoaging induced by chronic UVB irradiations, the strong anti-photoaging and anti-senescence properties of baicalin were directly linked to its effects on SASP. Additionally, baicalin modulated both the percentage of β-galactosidase-positive cells and the intracellular expression levels of p16, p21, and p53 in UVB-irradiated dermal fibroblasts [333]. Moreover, baicalin pre-treatment caused a significant decrease in the number of UVB-mediated DNA double-strand breaks in the chronically UVB-irradiated skin fibroblasts [333]. In vitro enriched-baicalein treatment also decreased the level of UVB-induced MMP-1 production and promoted type-1 procollagen synthesis in human dermal fibroblasts. In vivo, enriched-baicalein extract also caused a significant inhibition in UVB-induced wrinkle formation, epidermal thickening, and damage to collagen fibres in the mice skin [329]. The protective role of baicalein against UVB-induced photoaging has also been linked to its ability to block the radiation-induced cytosolic Ca^2+^ increase, thereby protecting the skin cells against UVB-induced MMP-1 expression and apoptosis in human dermal fibroblasts [334].

In a skincare study, baicalin treatment also provided an effective protection against UVA-induced photoaging features in dermal fibroblasts characterised by a reduction in telomere length, increased MDA and the expression of the enzyme MMP-1. These implied that the underlying mechanism of baicalin protection against UVA radiation is related to the inhibition of oxidative damage and regulation of the expression of senescence-related genes, including those encoding for p53, p66(Shc) and p16 [322]. In addition, *S. baicalensis* extract promoted the differentiation of epidermal keratinocytes and helped restoring the skin barrier function via the activation of a peroxisome proliferator-activated receptor, (PPAR)-α [335,336]. Furthermore, *S. baicalensis* extract appears to be a safe and effective cosmetic sunscreen, as it posesses a strong UVA-absorbing ability in the range of 320 to 400 nm [337]. Taken together, balcalin and balcalein can be classified as natural AOs with robust redox- and iron-homeostasis balancing properties. These multifunctional flavonoids are now finding their way into skincare products as suitable ingredients with strong anti-aging, anti-photoaging, rejuvenating and photoprotective properties.

### 4.3. Leontopodium alpinum

*Leontopodium nivale* ssp. *alpinum* (syn. *Leontopodium alpinum*), a perennial herb commonly known as Edelweiss, which has a long tradition in Alpine countries, is traditionally employed in folk medicine as an anti-inflammatory remedy. Different types of extracts and compounds derived from this plant, such as terpenoids (analogues of sesquiterpenes, bisabolenes), phenylpropanoids (phenolic acids, flavonoids, coumarins, lignans), fatty acids and polyacetylenes, were previously isolated from various parts of Edelweiss and have been found to possess a broad spectrum of pharmacological activities. Furthermore, the plant has known anti-inflammatory, antimicrobial, AO and chemoprotective effects. Edelweiss extract is mainly composed of leontopodic acid (55%). The compound has demonstrated significant ROS scavenging ability. Leontopodic acid was able to increase GPx activity in a human myeloid leukaemia cell line. Due to its significant AO efficacy, it could possibly be used as a supportive agent in the treatment of oxidative stress-related diseases and conditions [338].

Data from a panel for the preselection of natural photoprotective substances suggest leontopodic acid as one of the best promises for the complete natural topical prevention of photoaging and rejuvenation of photoaged skin. This is due to the fact that leontopodic acid is less susceptible to photodegradation and its SPF declined slower. The entirely ‘‘natural’’ approach to prevent photoaging and UV-related skin pathologies could, therefore, diminish the negative impact of synthetic sunscreens toward human health and the environment [283].

In vitro studies demonstrated the strong AO activity of *L. alpinum* callus culture extract in response to UVB treatment by suppressing inflammation and wrinkling. In vivo tests demonstrated that constant application of the extract on the face and skin tissues improved anti-periorbital wrinkles, skin elasticity, dermal density, and skin thickness compared with the placebo. Moreover, *L. alpinum* callus culture extracts upregulated genes encoding several keratin proteins, and also DDIT4, BNIP3, and IGFBP3 which are involved in the positive regulation of the developmental process, programmed cell death, keratinisation, and cornification by forming skin barriers, all of which provide many advantages to the human skin. Interestingly, the callus extract down-regulated stress-responsive genes, suggesting it as a promising agent for anti-aging cosmetics [339]. A concentrated ethanolic extract of culture homogenate, with leontopodic acid representing 55% of the total phenolic fraction, was characterised for its anti-inflammatory properties in primary human keratinocytes and endotheliocytes. This phenolic fraction showed remarkable sirtuin activating properties, which assumes its possible anti-inflammatory action through this molecular pathway. It also inhibited LPS-induced IL-6 and VCAM1 genes in HUVEC, as well as oxLDL-induced selective VCAM1 overexpression. Concentrated ethanol extract of *L. alpinum* callus cell cultures possessed remarkable anti-inflammatory properties at both transcriptional and translational levels in the cultured human skin keratinocytes and endothelial cells, thus confirming the ethnopharmacological use of the parental herb and its potential application for chronic inflammatory skin diseases [340].

Overall, the powerful AO, anti-inflammatory, anti-aging and anti-photoaging properties of *L. alpinum* extract support the view for its incorporation into skincare products and sunscreen formulations.

## 5. Conclusions

With a clear shift of the consumer market from synthetic compounds to natural-based and bio-inspired skincare and sunscreen ingredients, it is necessary to adopt a systematic approach to evaluate the effectiveness of current natural products in the market, in order to identify opportunities to improve the future of skincare and sunscreen formulations. The use of natural extracts with UV absorbing properties are also replacing gradually the synthetic compounds as sunscreen agents, due to a global demand for environmentally friendly and safe compounds lacking harmful effects on the sealife and individuals. To ascertain the photoprotective property of natural extracts, they should demonstrate consistent levels of active constituents in the extracts with appropriate stability, which remains a cause for concern. This is because the phytochemical profile of plant extracts can vary based on a series of biotic and abiotic factors, notably, latitude, season, soil, time of harvest, etc. Therefore, standardisation of the phytochemical profile remains a challenge. In addition, in extracts, often the biological functions are not fully explored which raises the necessity to undertake in-depth research for understanding the cell signaling and the exact mechanisms of actions of the compound. There is also a clear need for undertaking more clinical trials to determine the level of skin absorption, irritation, the genetic and phototoxicity profile, as well as the allergens content in different populations. Additionally, the use of natural compounds extracted from the plant extracts would require appropriate purification steps to decrease the toxicity caused by impurities due to poor extraction methodologies. The use of green chemistry may circumvent some of the toxicity issues. Furthermore, the use of green chemistry for the synthesis of bio-inspired molecules with higher stability than the parental counterparts may decrease significantly the problems of poor stability, as well as variabilities observed in the level and potency of active ingredients from different natural sources. Finally, for selecting the effective natural AOs for skin protection against premature intrinsic aging and extrinsic photoaging processes, it is necessary to take into consideration the dual influences of skin redox- and iron-homeostasis and, hence, the strength by which the AOs can restore the balance in redox- and iron-homeostasis in oxidative conditions. The general acceptance of these criteria will provide novel insights into skincare and photoprotection, leading to the launch of novel generations of safe, stable and powerful natural-based or bio-inspired bifunctional anti-aging and photoprotective ingredients with both redox- and iron-balancing properties.
